# Supplementary material for: The effect of social preference on academic diligence in adolescence
Source: R Soc Open Sci. 2019 Sep 11;6(9):190165. doi: 10.1098/rsos.190165 (PMC6774955; doi:10.1098/rsos.190165)
Supplement: Supporting information [file rsos190165supp1.docx]

**Supporting Information**

**Additional measures**

We included additional questionnaires (see Table S1 for means and standard error) to look at the relationship between performance on the task and measures of subclinical autistic traits (using the Autism-Spectrum Quotient (AQ). This questionnaire consists of 50 items and has good reliability for determining where any given individual sits on the autism spectrum [26]. Measures of social reward were collected using the Social Reward Questionnaire – Adolescent Version (SRQ-A) [27]. This questionnaire consists of five subscales: enjoyment of admiration, negative social potency, passivity, prosocial interactions and sociability. This is a valid, reliable measure of social reward in adults and adolescents. Academic diligence is also related to ‘grit’, defined as perseverance and a passion for long-term and challenging goals [17]. Therefore, we also included a standard questionnaire measure of ‘grit’, consisting of 12 items, where individuals are required to compare themselves to ‘most people’ on questions such as ‘I have overcome setbacks to conquer an important challenge’ [28]. We predicted that the tendency to spend more time looking at social photographs might be inversely related to autistic traits and positively related to admiration, prosocial interactions and sociability. We also predicted that time spent doing maths would be positive related to higher grit scores. However, none of these measures correlated with task performance or self-reported enjoyment ratings (all p>.05).

| **Table S1:** Questionnaire descriptive statistics | | | |
| --- | --- | --- | --- |
| Measure | Adolescents (mean, SD) | Adults (mean, SD) | p value, t test |
| WASI Matrices Raw | 27.12 (2.86) | 30.62 (3.86) | <.001 |
| AQ | 15.7 (6.08) | 19.46 (6.90) | .008 |
| SRQ Sociability | 5.50 (1.04) | 4.82 (1.23) | .007 |
| SRQ Prosocial | 6.5 0 (0.54) | 6.13 (0.64) | .009 |
| SRQ Admiration | 5.73 (0.80) | 5.79 (0.64) | .690 |
| GRIT | 2.89 (0.64) | 2.69 (0.58) | .113 |
| Note: Max score for AQ is 50, for SRQ subscales is 7 and 5 for grit. Adolescents N=43, Adults N=46 (44 for WASI Matrices). | | | |

**Switches**

We recorded the number of switches each participant made between the maths questions and looking at the show-reel of photographs, within each ADT condition. There was no significant main effect of age group (*F*(1,89) =0.581, *p*=0.448, η^2^=.006) or condition (*F*(1,89)=.0.077, *p*=.782, η^2^=.001) on the number of switches taken. There was also no significant interaction between age group and condition (*F(*1,89) =0.566, *p*=.454, η^2^=.006). Controlling for autistic traits, social reward and grit did not affect the significance of these findings. In each case there were no significant effects (p<.05)

**Maths problems completed prior to switching**

|  | Social Condition | | Non-Social Condition | |
| --- | --- | --- | --- | --- |
|  | Adolescents | Adults | Adolescents | Adults |
| Mean | 51.04 | 79.39 | 56.38 | 72.09 |
| SD | 68.72 | 88.01 | 70.06 | 101.6 |
| Table S2: Mean and standard deviations of the number of maths problems completes before the first switch from maths to pictures, by condition. | | | | |

There was no main effect of condition (*F*(1,89)=0.01, *p*=9.20, η^2^= .00) or age group (*F*(1,89)=2.30, *p*=.133, η^2^= .025) on the number of maths problems completed prior to the first switch to look at the pictures. There was no interaction between age group or condition (*F*(1,89)=0.42, *p*=.52, η^2^= .01) (see table S2 for descriptive statistics).

**Enjoyment rating for maths**

All 45 adolescent participants provided an enjoyment rating for the maths in both conditions, but 4 of the 46 adult participants did not provide a rating in both conditions and were therefore not entered into the analysis. There was a significant main effect of age group (*F*(1,85)= 16.1, *p*=.001, η^2^=.159) on maths enjoyment ratings. In the social condition, adolescents reported enjoying the maths significantly less than did the adults (*t*(85)= -3.723, *p*=.004). Similarly, in the non-social condition, adolescents reported enjoying the maths significantly less than did the adults (*t*(85)=-3.631, *p*=.004). There was no main effect of condition (*F*(1,85)= 0.689, *p*= .409, η^2^= .008) on maths enjoyment ratings, nor was there was a significant interaction between age group and condition (*F*(1,85)= 0.03, *p*= .863, η^2^= .001) (Figure S1; Table S1 for means and standard error). Controlling for autistic traits, social reward and grit did not affect the significance of these findings. In each case there was a significant main effect of age (p<.05) and all other effects were non-significant (p>.05).

**
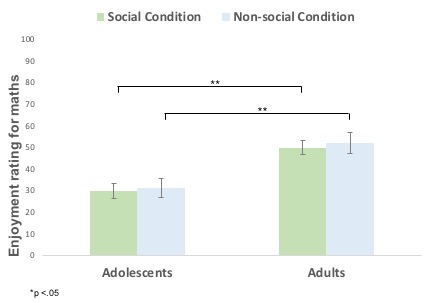
**

**Figure S1:** Enjoyment ratings

Mean enjoyment ratings for the maths in each condition, for adolescents and adults. Asterisks indicate Bonferroni corrected P values.

**Distributions of time spent looking at the pictures**

**Figure S2:** Distribution of time spent looking at non-social pictures in the non-social ADT


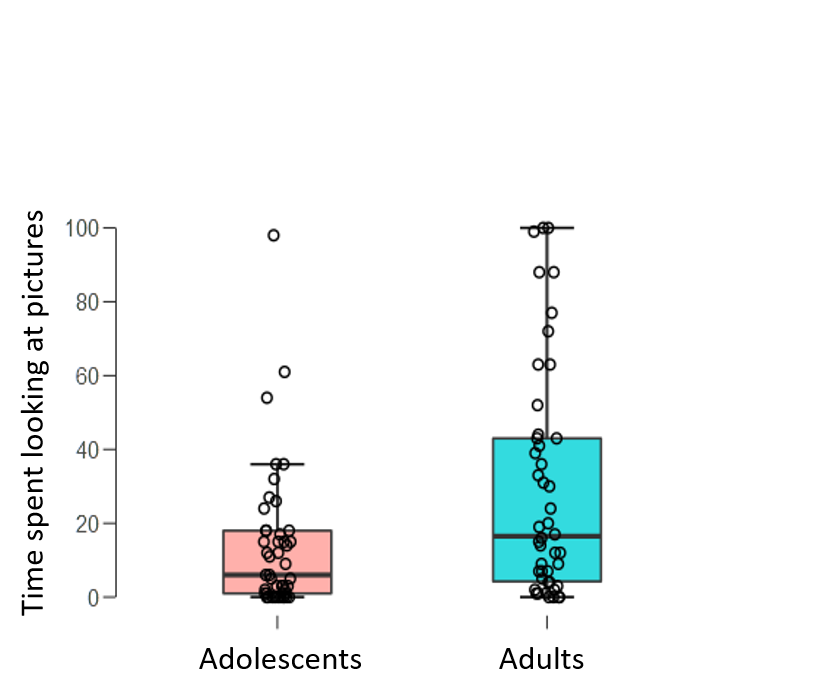


**Figure S3:** Distribution of time spent looking at social pictures in the social ADT


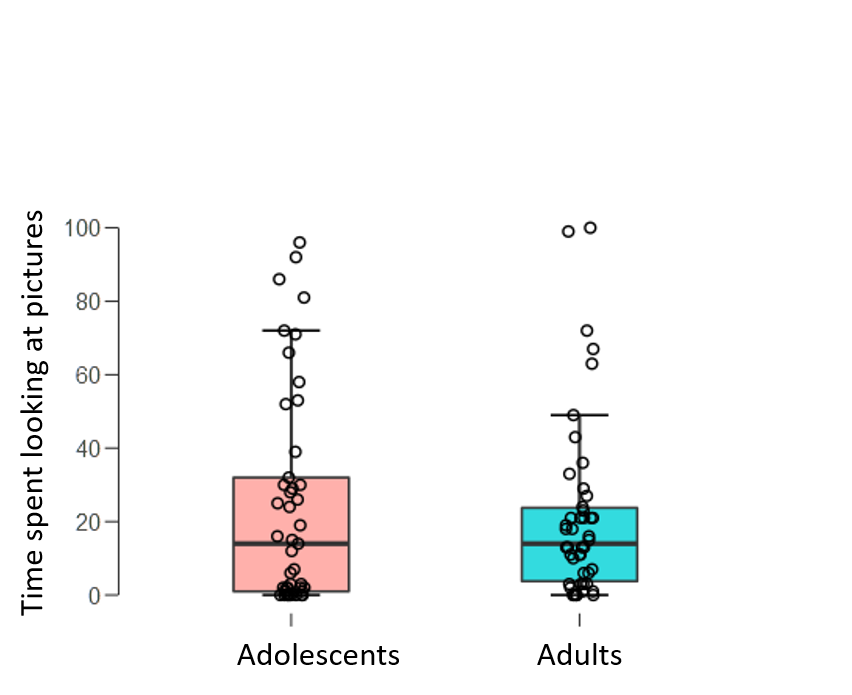


**Additional references:**

26. Baron-Cohen S, Wheelwright S, Skinner R, Martin J, Clubley E. 2001 The autism-spectrum quotient (AQ): evidence from Asperger syndrome/high-functioning autism, males and females, scientists and mathematicians.
J. Autism Dev. Disord. 31, 5–17. (doi:10.1023/ A:1005653411471)

27. Foulkes L, Neumann CS, Roberts R, McCrory
E, Viding E. 2017 Social Reward Questionnaire—Adolescent Version and its association with callous–unemotional traits.
R. Soc. open sci. 4, 160991. (doi:10.1098% 2Frsos.160991)

28. Duckworth AL, Peterson C, Matthews MD, Kelly
DR. 2007 Grit: perseverance and passion for long-term goals. J. Pers. Soc. Psychol. 92, 1087. (doi:10.1037/0022-3514.92.6.1087)
